# CitH3: a reliable blood biomarker for diagnosis and treatment of endotoxic shock

**DOI:** 10.1038/s41598-017-09337-4

**Published:** 2017-08-21

**Authors:** Baihong Pan, Hasan B. Alam, Wei Chong, James Mobley, Baoling Liu, Qiufang Deng, Yinjian Liang, Yanming Wang, Eric Chen, Tianbing Wang, Muneesh Tewari, Yongqing Li

**Affiliations:** 1 Department of General Surgery, Xiangya Hospital, Changsha, Hunan China; 2 Department of Surgery, University of Michigan Hospital, Ann Arbor, MI USA; 3 The First Hospital, China Medical University, Shenyang, China; 4 Cayman Chemical, Ann Arbor, MI USA; 5 Department of Biochemistry and Molecular Biology, Penn State University, University Park, PA USA; 6 Department of Trauma and Orthopedic Surgery, Peking University People’s Hospital, Beijing, China; 7 Department of Internal Medicine, University of Michigan, Ann Arbor, MI USA

## Abstract

Current biomarkers for sepsis are limited by their non-specificity, short half-life, and insensitive response to therapy. Recently, we have demonstrated that citrullinated histone H3(CitH3) is released into the blood from neutrophil extracellular traps(NETs) in response to severe infection, and CitH3 may be a potential biomarker for sepsis. In the present study, we found that NET components were released in mouse models of both lipopolysaccharide(LPS)-induced shock (LPSS) and hemorrhagic shock (HS). To further quantify CitH3 in the NETs, we established a CitH3 specific enzyme-linked immunosorbent assay. Circulating CitH3 was found to be elevated only in LPSS but not in HS. Importantly, blood CitH3 was detected 30 minutes after LPS insult, and remained elevated for 24 hours (period of the highest mortality). Treatment of endotoxic mice with YW3-56, a peptidylarginine deiminase-2/4 inhibitor, significantly diminished levels of CitH3 in the blood. Interleukin-1β did not respond to LPS early, and interleukin-1β and interleukin-6 fluctuated although they responded to treatment. Procalcitonin reacted to LPS insult late. Compared to CitH3, these biomarkers were non-specifically induced in LPSS and HS. Collectively, our results demonstrate that YW3-56 protects animals from LPSS, and CitH3 is a reliable biomarker due to its early appearance, specificity, duration, and response to therapeutic intervention.

## Introduction

Sepsis is a leading cause of death in the intensive care units^1^. Based on the latest consensus, sepsis is defined as life-threatening organ dysfunction induced by a dysregulated host response to infection^2^. During the early stage of sepsis, the host experiences a critical pathological event called infectious inflammation, the inflammatory response that is also seen in traumatic injury (*i*.*e*., non-infectious inflammation). Given the fact that sepsis often accompanies other injurious hits to the body such as trauma, hemorrhagic shock, major surgery etc.^3^, this shared inflammatory response makes early diagnosis of sepsis challenging. Novel strategies are needed to seek a biomarker to detect sepsis early and distinguish it from the non-infectious inflammation caused by trauma. This distinction would allow prompt and appropriate use of antibiotics in sepsis, while preventing their misuse in non-infectious inflammation.

Strong evidence has shown that citrullination of histone H3 (CitH3) is catalyzed by peptidylarginine deiminase (PAD), triggering neutrophil extracellular trap (NET)^4^ formation. The process of NET formation, referred to as NETosis^5^, is considered a host defense mechanism^6^. Excessive NETosis is a hallmark of sepsis^7^. NETosis can result in the release of nuclear contents into the extracellular milieu^8^, mainly histones and DNA. It is unknown whether CitH3 is released via a sepsis-related mechanism or whether it could be a specific biomarker for diagnosis of and treatment for sepsis. To date, five isozymes of PAD (1, 2, 3, 4 and 6) have been identified^9^. Of these PADs, only nuclear-localized PAD2^10^ and PAD4^11^ can citrullinate histone H3 and induce NETosis. It remains unclear whether inhibition of both PAD2 and PAD4 could decrease the levels of CitH3 in the blood.

YW3-56 is a compound originally used for treatment of cancer that can inhibit PAD2 (IC50: 0.5–1 µM) and PAD4 (IC50: 1–2 µM)^12^. We have recently found that treatment of septic mice with this PAD2/PAD4 inhibitor can significantly improve survival in a model of lethal lipopolysaccharide (LPS)-induced shock (LPSS) (manuscript submitted for publication). We have demonstrated using Western blot that CitH3 is released into the circulation during the early stages of LPS-induced shock (3 hours after peritoneal injection of LPS), and CitH3 levels in the circulation are significantly associated with the severity of shock^13^. Moreover, neutralization of circulating CitH3 with specific anti-CitH3 antibody is protective in septic mice^14^. Based on these findings, we hypothesized that CitH3 could be a reliable endotoxemia-specific biomarker compared to the existing clinical biomarkers, such as procalcitonin (PCT) and pro-inflammatory cytokines (e.g., interleukin-1β (IL-1β) and interleukin-6 (IL-6)).

It has been suggested that an ideal septic biomarker should have the following characteristics^15–18^: (1) short time of induction after a bacterial stimulus, (2) specificity (e.g., positive for sepsis and negative for non-infectious inflammation), and (3) long half-life (to be detected in circulation). However, it remains to be determined whether CitH3 possesses these characteristics, and whether it is responsive to therapeutic intervention. Therefore, using a mouse model of LPSS in the current study, we aimed to quantify the levels of circulating CitH3 by enzyme-linked immunosorbent assay (ELISA) and determine the effects of YW3–56 on CitH3 in the blood at multiple time points. Moreover, we intended to assess advantages of CitH3 as a reliable specific biomarker for endotoxic shock, compared to PCT, IL-1β, and IL-6 in both mouse models of LPSS and hemorrhagic shock (HS).

## Results

### NETs formation is induced by both LPS and HS

**Figure 1:**
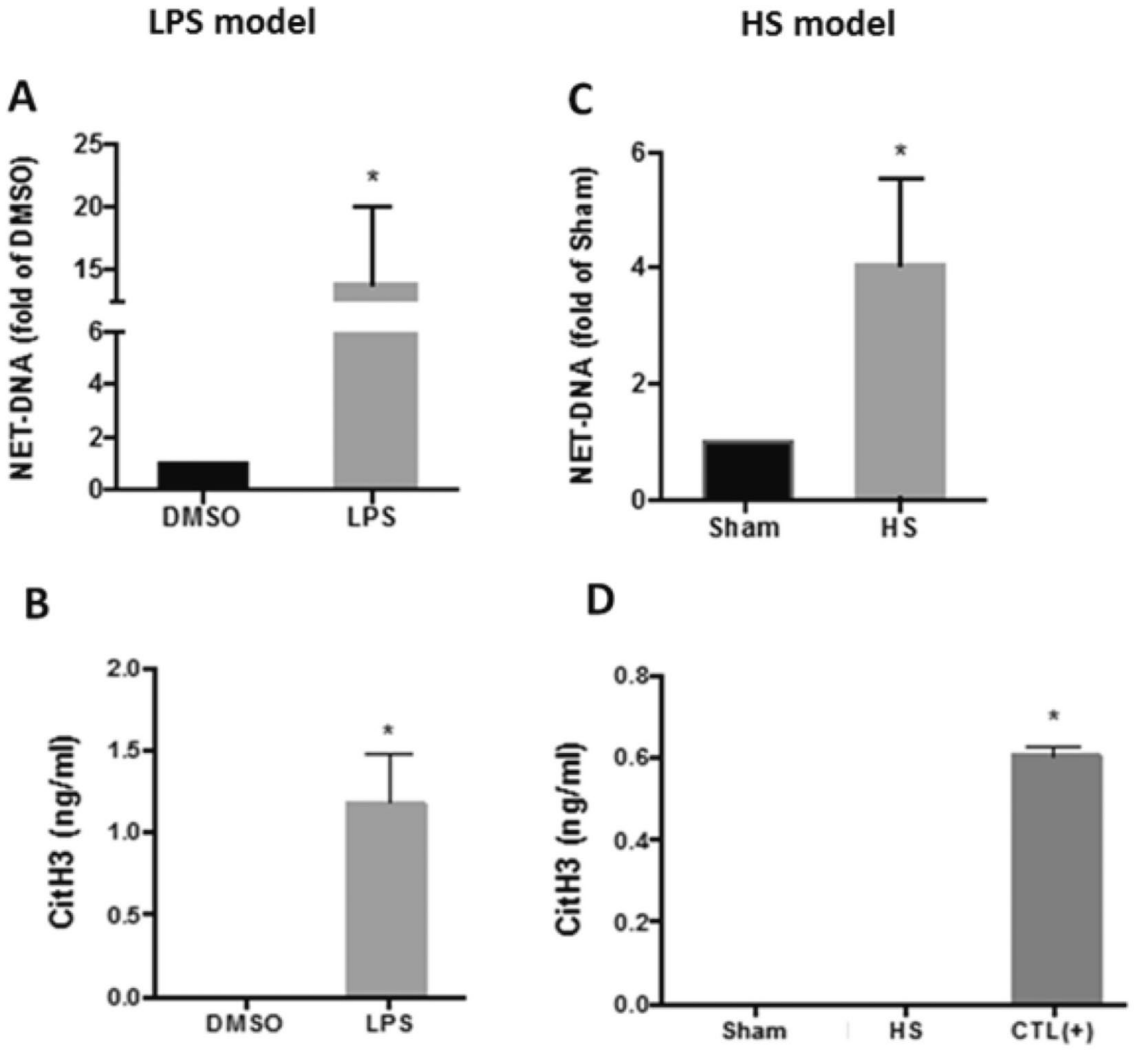
NETs formation is induced by both LPS and HS. Animals were intraperitoneally administrated LPS (35 mg/kg) (**A** and **B**) or received hemorrhage (30% blood loss) without any treatment (**C** and **D**). Animals from Sham group received all procedures except cannulation and hemorrhage. CitH3 protein serves as a positive control in panel D. Blood samples were harvested 12 hours post LPS or HS. The double stranded DNA (**A** and **C**) was dramatically elevated after LPS and HS insult compared to DMSO and Sham group, respectively. CitH3 was significantly increased after LPS insult (**B**) while was un-detectable after HS (**D**). Data are shown as mean ± standard deviation (SD) (n = 4–6/group). CitH3: Citrullinated histone H3; LPS: Lipopolysaccharide; DMSO: dimethyl sulfoxide; HS: hemorrhagic shock; NET: neutrophil extracellular trap; DNA: deoxyribonucleic acid. CTL (+): positive control.

We have previously demonstrated that NETs are the source of circulating CitH3 in mouse model of LPSS^13^. Given the fact that NETs are chromatin with a web-like structure^6^, we measured the circulating double stranded DNA (dsDNA) as an indicator of NETs formation in LPSS and HS. In LPSS, animals were intraperitoneally (i.p.) administered LPS (35 mg/kg) or DMSO (vehicle control). HS mice were subjected to 30% blood loss^13^. Blood samples were harvested at 12 hours after LPS or HS. The levels of dsDNA and CitH3 in the blood serum were measured. The concentrations of dsDNA (Fig. 1A and C) were dramatically elevated after LPS or HS insult, compared to the DMSO group (1486.8 ± 671.2 pg/ml vs 141.4 ± 40.2 pg/ml, *p* = 0.0135) and Sham group (437.2 ± 163.4 pg/ml vs 74.1 ± 24.1 pg/ml, *p* = 0.0185), respectively. Circulating CitH3 was significantly increased after LPS injection (Fig. 1B) (1.18 ± 0.3 ng/ml vs 0 ± 0 ng/ml, *p* = 0.0025) while the CitH3 was un-detectable after HS (Fig. 1D). These results indicate that NET formation occurs in both LPSS and HS, though HS does not induce the same type of NETs as LPSS does.

### Different treatments (LPS, LPS + YW3-56, or HS) affect blood levels of CitH3 in LPSS and HS

**Figure 2:**
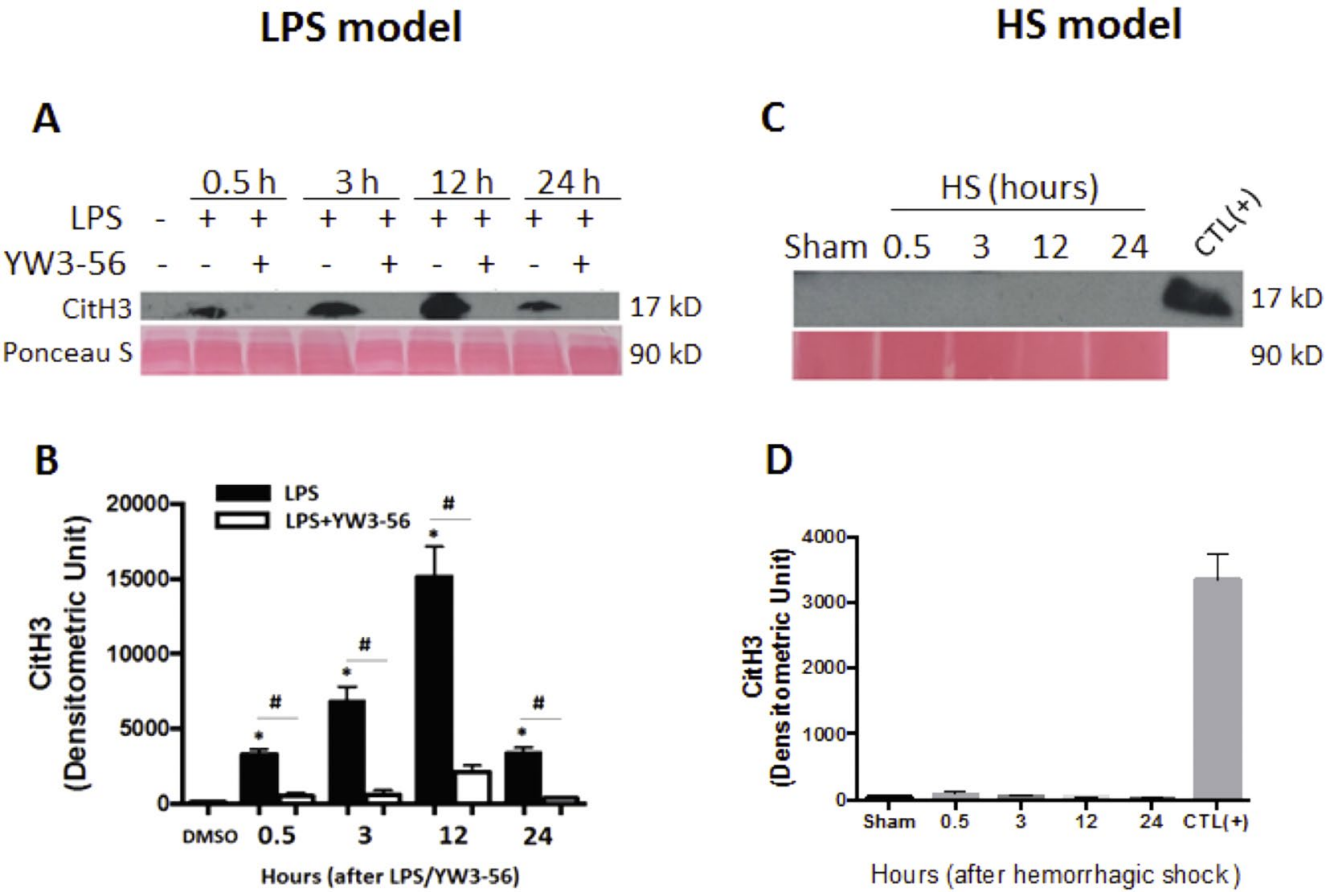
Effect of different treatments (LPS, LPS + YW3-56, or HS) on blood levels of CitH3 in mouse models of LPSS and HS. Mice were intraperitoneally injected with LPS (35 mg/kg) in presence or absence of YW3-56 (**A**) or received hemorrhage (30% blood loss) (**C**). Animals from Sham group received all procedures except cannulation and hemorrhage. Blood was harvested at 0.5, 3, 12 and 24 hours after treatments. Circulating CitH3 was detected by Western blotting. Ponceau S stain was performed as loading control. Densitometry units were measured by Image J (**B** and **D**). Serum from LPS treated mice (35 mg/kg, 12 hours post LPS) served as positive control (CTL) (**C**). Data are shown as mean ± SD, n = 3/group. CitH3: Citrullinated histone H3; LPS: Lipopolysaccharide; HS: Hemorrhagic shock; DMSO: dimethyl sulfoxide.

To determine the specificity of CitH3 for LPSS, two mouse models were used. In the LPSS model, mice were injected i.p. with LPS (35 mg/ml) in the absence or presence of YW3-56 (5 mg/kg) (Fig. 2A and B). In the HS model, mice were subjected to 30% blood loss without any resuscitation (Fig. 2C and D). Based on the survival result in this study (data submitted for publication separately), the majority of the animals died within 24 hours following the LPS insult. Therefore, we decided to measure levels of CitH3 in the blood for a duration of 24 hours post LPS. Serum samples were collected at 0.5, 3, 12, and 24 hours after the treatment, and Western blot was performed with an anti-CitH3 antibody (Fig. 2A and C). As shown in Fig. 2A, no circulating CitH3 was detected in the DMSO group. Serum levels of CitH3 were elevated as early as 0.5 hour after LPS injection, increased further at 3 hours, reached a peak at 12 hours, and were sustained for 24 hours compared to the DMSO group (3294.1 ± 349.9 vs 89.8 ± 48.8, *p* = 0.0026; 6792.7 ± 1014.3 vs 89.8 ± 48.8, p < 0.0001; 15147.3 ± 2004.3 vs 89.8 ± 48.8, *p* < 0.0001; 3346.4 ± 401.8 vs 89.8 ± 48.8, *p* = 0.0022; respectively) (Fig. 2B). YW3-56 treatment significantly decreased LPS-induced CitH3 in the blood at all four time-points compared to the LPS group (3294.1 ± 349.9 vs 45.2 ± 29.6, *p* = 0.0089; 6792.7 ± 1014.3 vs 49 ± 16.4, *p* < 0.0001; 15147.3 ± 2004.3 vs 436.6 ± 8.5, *p* < 0.0001; 3346.4 ± 401.8 vs 16.3 ± 46, *p* = 0.0049; respectively). In the HS model, no CitH3 was detected in the blood by Western blotting at any time-points. Serum of mice treated with LPS for 12 hours served as the positive control (Fig. 2C and D). The results suggest that LPS, but not HS, increases CitH3 in the blood, and presence of CitH3 can be measured for many hours following an endotoxic insult.

### CitH3 is a sensitive and long-lasting biomarker compared to PCT and pro-inflammatory cytokines in endotoxic shock

**Figure 3:**
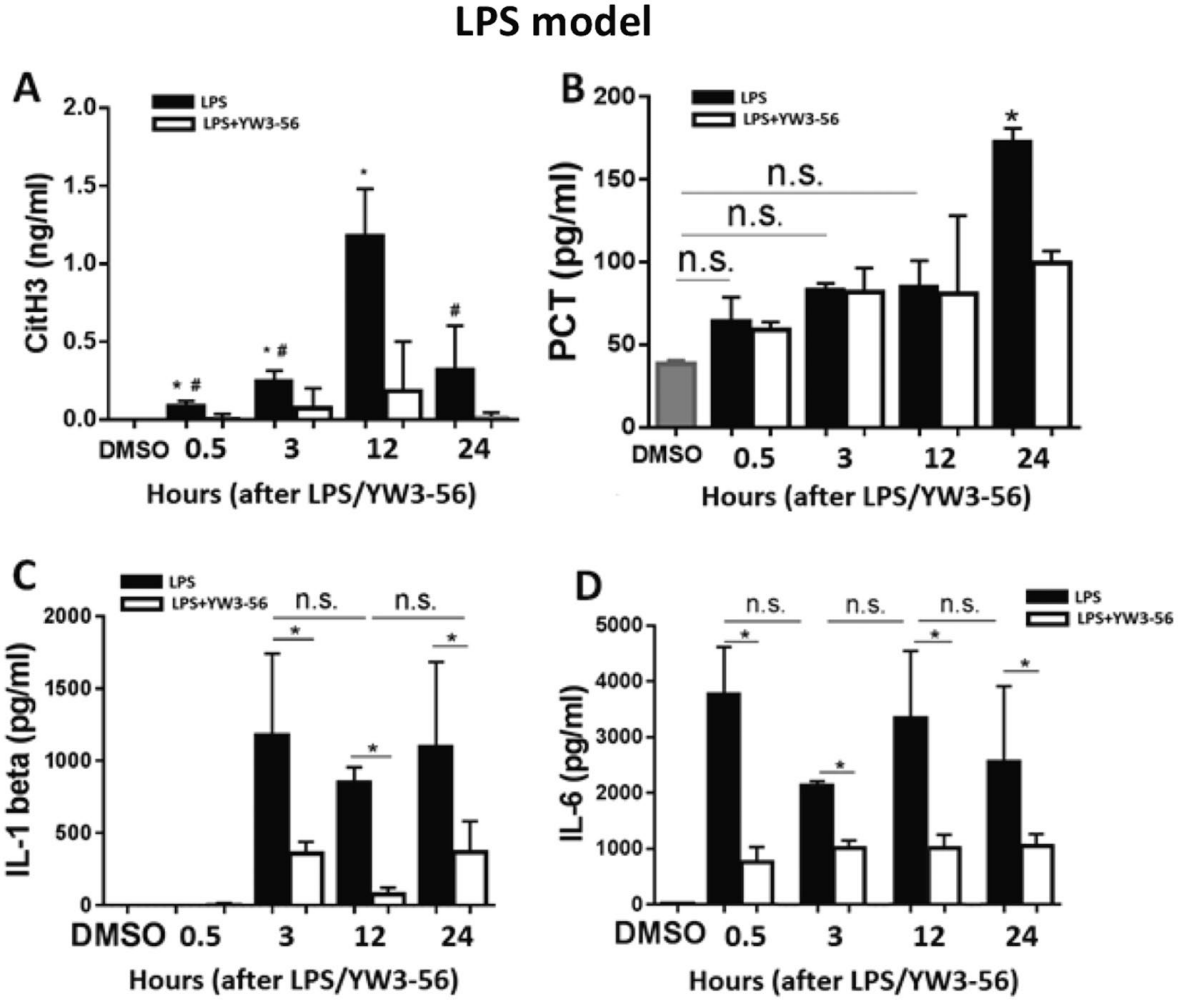
CitH3 is a sensitive and long-lasting biomarker compared to PCT, IL-1β and IL-6 in LPSS. Mice were intraperitoneally administrated LPS (35 mg/kg) with or without YW3-56. Sera were prepared at 0.5, 3, 12 and 24 hours after LPS for measurement of CitH3 (**A**), PCT (**B**), IL-1β (**C**) and IL-6 (**D**) by ELISA. (**A**) Circulating CitH3 was detected quickly (0.5 hour), accumulated maximally at 12 hours, and sustained for 24 hours. YW3-56 significantly attenuated LPS-induced increase of CitH3. *p < 0.05 compared to DMSO, #*p* < 0.05 compared to 12 hours post LPS. (**B**) LPS could not induce a significant increase of PCT in blood in the early stage of endotoxic shock (0.5, 3 and 12 h) till 24 hours compared to DMSO group (*p* < 0.0001). (**C** and **D**) Pro-inflammatory cytokines irregularly fluctuated after LPS administration (IL-1β & IL-6). They did not respond to LPS early (IL-1β), although they were attenuated by YW3-56 treatment. Data was shown as mean ± standard deviation (SD) (n = 3–5/group). IL-1β: interlukine-1β; IL-6: interlukine-6; CitH3: Citrullinated histone H3; LPS: Lipopolysaccharide; DMSO: dimethyl sulfoxide. PCT: procalcitonin; LPSS: LPS-induced endotoxic shock.

In addition to the analysis of CitH3 in the blood by Western blot, we developed a “sandwich” indirect enzyme-linked immunosorbent assay (ELISA) to more precisely measure the concentration of circulating CitH3. Mice were administered LPS (35 mg/kg, i.p.) with or without YW3-56 (5 mg/kg) treatment. Sera were collected at 0.5, 3, 12, and 24 hours after LPS injection. The ELISA results were consistent with the Western blot results (Fig. 2A). As shown in Fig. 3A, no CitH3 was detected in the DMSO group. LPS induced a significant increase of CitH3 in the blood at 0.5, 3, 12, and 24 hours compared to DMSO group (87 ± 32.1 vs 0 ± 0 pg/ml, *p* = 0.0095; 245 ± 68.7 vs 0 ± 0, *p* = 0.0022; 1178 ± 303.3 vs 0 ± 0, *p* = 0.0025; 318 ± 283.8 vs 0 ± 0, *p* = 0.124; respectively). Treatment with YW3-56 dramatically diminished the increase at all time-points in comparison with the LPS group (13 ± 23.1 pg/ml vs 87 ± 32.1 pg/ml, *p* = 0.0326; 73 ± 127 pg/ml vs 245 ± 68.7 pg/ml, *p* = 0.0498; 183 ± 317.5 pg/ml vs 1178 ± 303.3 pg/ml, *p* = 0.0172; 17 ± 28.9 pg/ml vs 318 ± 283.8 pg/ml, *p* = 0.141; respectively).

In order to compare CitH3 to the existing biomarkers, PCT (Fig. 3B), IL-1β (Fig. 3C) and IL-6 (Fig. 3D) were measured by ELISA in the blood samples. The concentration of PCT in the DMSO group was 38.4 pg/ml. LPS did not induce a significant increase in PCT until the terminal stage (24 hours post LPS) compared to the DMSO group (64.1 ± 14.3 vs 38.4 ± 2.4, *p* = 0.7334; 83.1 ± 4.1 vs 38.4 ± 2.4, *p* = 0.1385; 84.9 ± 15.9 vs 38.4 ± 2.4, *p* = 0.112; 172.5 ± 8.4 vs 38.4 ± 2.4, *p* < 0.0001; respectively). YW3-56 dramatically decreased PCT level at the 24 hour time point compared to the LPS-only group (99.5 ± 7.2 vs 172.5 ± 8.4, *p* = 0.0033) (Fig. 3B). The pro-inflammatory cytokine IL-1β did not respond to LPS insult early (0.5 hours). Both IL-1β and IL-6 were highly variable after LPS injection, though they responded to YW3-56 treatment (Fig. 3C and D). The results suggest that CitH3 is a better candidate biomarker for endotoxic shock due to its early appearance, sustained presence in the circulation, and sensitive response to therapeutic intervention.

### CitH3 is a specific biomarker that can distinguish LPSS from HS

**Figure 4:**
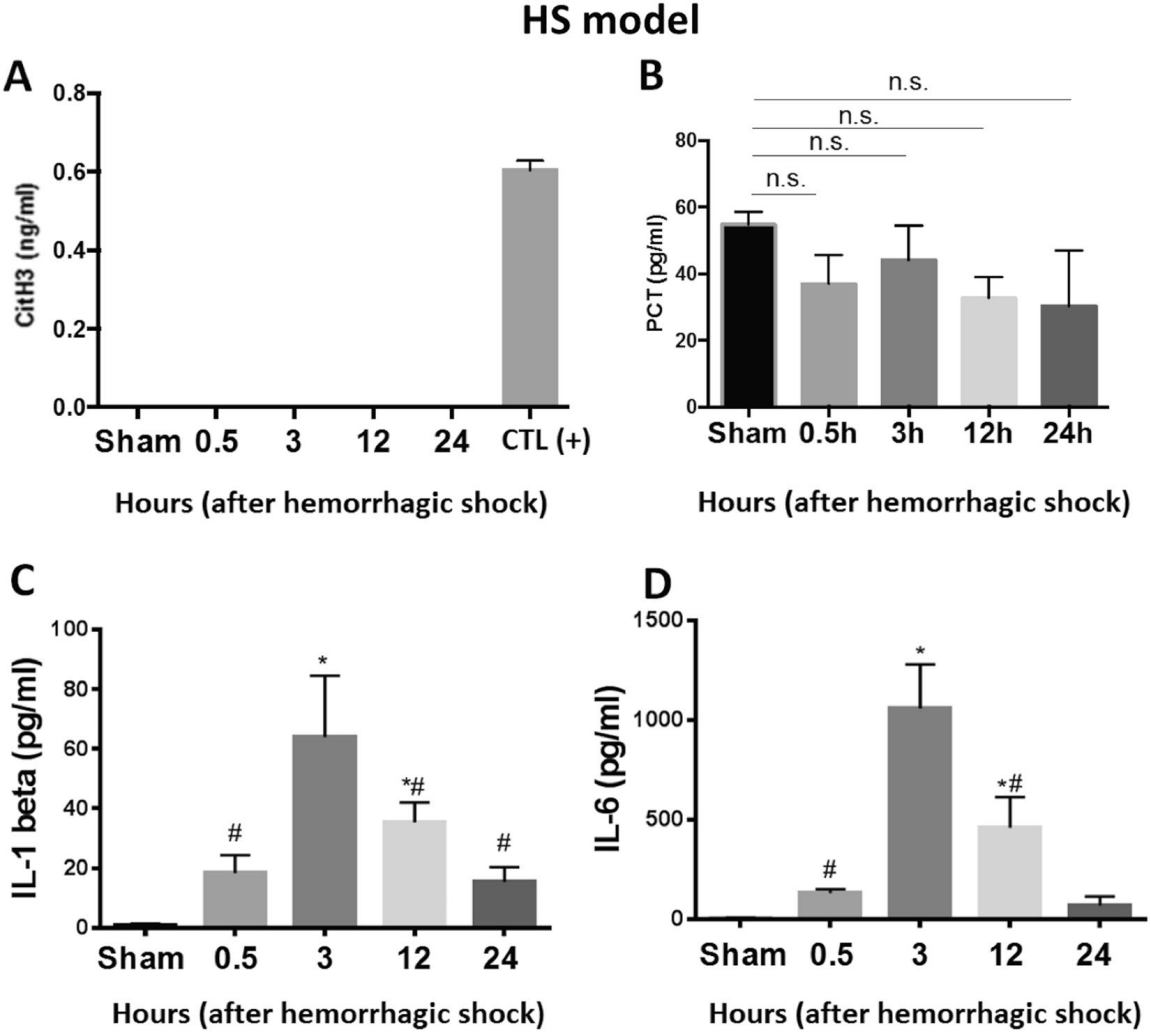
CitH3 is a specific biomarker to distinguish LPSS from HS. Mice were subjected to HS (30% hemorrhage without resuscitation) and blood samples were collected at 0.5, 3, 12, and 24 hours after HS for detection of CitH3 (**A**), PCT (**B**), IL-1β (**C**) and IL-6 (**D**) by ELISA. Animals from Sham group received all procedures except cannulation and hemorrhage. CitH3 protein serves as a positive control in panel A. No circulating CitH3 was detectable by ELISA. HS did not alter the blood concentration of PCT compared to Sham group. Pro-inflammatory cytokines (IL-1β & IL-6) elevated greatly compared to Sham. Data are shown as mean ± standard deviation (SD). (n = 3–5/group). IL-1β: interlukine-1β; IL-6: interlukine-6; CitH3: Citrullinated histone H3; LPS: Lipopolysaccharide; DMSO: dimethyl sulfoxide. PCT: procalcitonin; HS: hemorrhagic shock. CTL (+): positive control.

To determine the diagnostic specificity of CitH3 as well as other biomarkers’, all markers were measured in the HS model. Mice were subjected to HS (30% hemorrhage without resuscitation) and blood samples were collected at 0.5, 3, 12, and 24 hours after HS for detection of CitH3 (Fig. 4A), PCT (Fig. 4B), IL-1β (Fig. 4C) and IL-6 (Fig. 4D) by ELISA. Animals from the Sham group were subjected to all procedures except cannulation and hemorrhage. CitH3 protein served as a positive control in Fig. 4A. Similar to the results in Fig. 2B, circulating CitH3 was not detectable by ELISA (Fig. 4A). Hemorrhage did not alter the concentration of PCT in the serum at any of the four time points compared to the Sham group (37 ± 8.7 vs 54.7 ± 3.8, *p* = 0.2301; 44.1 ± 10.2 vs 54.7 ± 3.8, *p* = 0.6801; 32.7 ± 6.2 vs 54.7 ± 3.8, *p* = 0.2301; 30.2 ± 16.8 vs 54.7 ± 3.8, *p* = 0.2301, respectively) (Fig. 4B). In agreement with publications by other groups^19, 20^, our data showed that hemorrhagic shock induced a significant increase in IL-1β (18.4 ± 6 vs 0.8 ± 0.7, *p* = 0.0071; 64 ± 20.6 vs 0.8 ± 0.7, *p* = 0.0001; 35.4 ± 6.6 vs 0.8 ± 0.7, *p* = 0.0137; 15.4 ± 5.0 vs 0.8 ± 0.7, *p* = 0.0075, respectively) and IL-6 (131.9 ± 20.4 vs 6.8 ± 1.3, *p* = 0.0005; 1.59.8 ± 221.1 vs 6.8 ± 1.3, *p* < 0.0001; 460.5 ± 154.3 vs 6.8 ± 1.3, *p* = 0.0075; 15.4 ± 5.0 vs 69.6 ± 46.4, *p* = 0.079, respectively), compared to the Sham group (Fig. 4C and D). The results indicate that circulating CitH3 exclusively appears in LPSS but not in HS, while pro-inflammatory cytokines respond to both infectious and non-infectious stimuli.

## Discussion

In the present study, we have demonstrated that NETs were generated in mouse models of both LPSS and HS. However, CitH3 in the blood were detected only in the LPSS, and not in HS mice. Circulating CitH3 appeared early, lasted for many hours, and responded to therapeutic intervention, which is a combination of characteristics that is in contrast to other conventional biomarkers (PCT, IL-1β, and IL-6) in circulation. To the best of our knowledge, this is the first study to quantify blood CitH3 and compare it with PCT and other cytokines using ELISA method.

This study offers evidence that CitH3 matches the characteristics of an ideal septic biomarker compared to other existing biomarkers^15–18^. First, we showed that CitH3 appears in blood quickly after endotoxin insult (*i*.*e*., within 30 minutes). Such a short induction time facilitates an early diagnosis of endotoxic shock. It has been known that there are three processes that lead to NET formation at a different rate^21^. The fastest one involves indirect cell-mediated activation. Under flow conditions, LPS-mediated activation of platelets binds to neutrophils, which induces release of neutrophil NETs within minutes^7^. In the second process of NET formation, leukocytes release their mitochondrial DNA to form NETs within 30 minutes after LPS injection^22^. In the third and slowest, neutrophils undergo nuclear de-condensation and cell rupture after exposure to microbes, resulting in NET formation in a maximum of 2-3 hours^23^. In the current study, we did not determine which process of NET formation is involved in elevation of CitH3. However, all the three models of NET formation (even the slowest) happen at a relatively quick speed, making early detection possible.

The second strength, that distinguishes CitH3 from the other biomarkers, lies in its specificity for LPSS. There are two main types of acute inflammatory responses, the infectious type such as LPSS, and the non-infectious type such as HS. Although we showed both kinds of the inflammatory responses trigger NETosis (Fig. 1), the actual molecular pathways are still unknown. Research studies have shown that PADs are key enzymes involved in NET formation^11, 24^. They mainly catalyze protein citrullinations. Of all isozymes of the PAD family, PAD2 and PAD4 are found to citrullinate histones at different arginine (R) residues^10, 25^. Recently, we revealed that CitH3 is released into the supernatant of HL-60 cells^13^, a neutrophil-like cell line^26^, after LPS stimulation. *In vivo*, circulating CitH3 was detected 3 hours post-LPS and its levels were significantly associated with the severity of endotoxic shock^13^. In the present study, we demonstrated that CitH3 was detected in the blood within 30 min after intraperitoneal injection of a large dose of LPS. However, CitH3 was not found in the blood of mice with HS at any of the time points. These results suggest that CitH3 satisfies a critical criterion for an ideal biomarker - specificity. This feature can allow us to differentiate sepsis from non-infectious inflammation.

Thirdly, the fact that CitH3 is detectable in the blood for 24 hours post LPS indicates that CitH3 has a relatively long half-life. This long detection period of CitH3 is a great clinical advantage, which allows for repeated measurements of the biomarker for diagnosis and prognosis of endotoxic shock. In the current study, we found that inhibition of PAD2/PAD4 with YW3-56 significantly improved survival while it diminished or abolished circulating CitH3, suggesting that blood levels of CitH3 could also reflect the drug’s effect. Furthermore, a good biomarker should be predictive. Recently, we have reported that an early increase in circulating CitH3 protein is associated with high lethality in a mouse model of LPS-induced shock, and that levels of CitH3 in blood can predict the outcome of the endotoxic shock^13^. Our results, therefore, add more value to CitH3 biomarker.

Finally, we showed that CitH3 is a better biomarker than PCT and pro-inflammatory cytokines. PCT has been considered the most promising sepsis biomarker in clinical use^27^. For the purposes of diagnosis and prognosis in critical care, PCT has an advance over other traditional markers of sepsis. However, PCT is not widely adopted by clinicians due to its non-specificity^28^. In our current mouse models, we found that PCT did not respond to LPS injection until 24 hours post shock, at which point most the mice were dying. Also PCT was not specific because of its appearance in both LPSS and HS.

It is not fully understood how signals transmitted through intracellular signal transduction pathway pass to nucleus, where histone H3 is citrullinated and NET is initiated. So far, there are at least two NETosis mechanisms suggested: vital (infectious) NETosis and suicidal (non-infectious) NETosis^29^. Suicidal NETosis^6^ classically occurs following stimulation by phorbol 12-myristate 13-acetate (PMA) or cytokine-induced neutrophil chemoattractant 1 (CINC-1, in rat)/interleukin-8 (IL-8, in human) through activation of protein kinase C and the raf-mitogen-activated protein kinase (MEK)-extracellular signal-regulated kinase (ERK) pathway. NADPH and myeloperoxidase (MPO) are required. It takes over 120 minutes for intracellular NET formation and the neutrophil outer membrane to rupture. By contrast, vital NETosis occurs after direct microbial exposure and lipopolysaccharide, and takes approximately less than 30 minutes^29^. It has been reported that different stimuli differ in their capacity to initiate suicidal NETosis and vital NETosis^29^. For example, PMA activates protein kinase C (PKC)α, and thus inhibits PAD4; whereas calcium ionophores, activate PKCζ, and thus PAD4^30^. We did not intensively investigate the mechanism involved in the current study. Our results, however, support the fact that activation of PADs is essential in the production of CitH3 and NET formation.

A good biomarker should be predictive. Our previous study revealed that blood levels of CitH3 are significantly associated with severity of LPS-induced shock (Li *et al*. Surgery. 2011; 150:442–451), suggesting that CitH3 is useful not only for diagnosis but also for prognosis of endotoximia.

This study has some limitations to be acknowledged. We performed experiments in a mouse model of LPSS, and compared the data to those in a mouse HS model. The LPSS model reflects a Gram negative bacterial infection, and does not cover Gram positive bacterial infections. Hemorrhagic shock, although severe, was not associated with any other traumatic injuries. In addition, the present experiments were essentially designed for a proof-of-concept study. We focused on CitH3 as a biomarker and treated the animals with PAD inhibitor YW3-56 instantaneously with LPS, in order to compare CitH3 to some existing sepsis biomarkers. Clinical study is currently ongoing to determine whether CitH3 can serve as a specific biomarker to diagnose patients with sepsis and distinguish sepsis from other types of severe non-infectious inflammation in clinical practice.

In conclusion, we have established a novel and specific indirect ELISA (more sensitive than direct ELISA) to quantitatively measure CitH3. With this quantitative measurement, we have accurately assessed circulating CitH3 levels in mouse models of LPSS and HS. Compared with serum PCT, IL-1β and IL-6, CitH3 is more responsive to endotoxemia. Our results suggest that CitH3 is a reliable biomarker for endotoxic shock due to its early appearance, high specificity, long half-life, and responsiveness to therapeutic intervention. CitH3 could potentially diagnose sepsis/septic shock and predict outcome of this life-threatening illness in the future.

## Methods

### Animals

All research was conducted in compliance with the guidelines approved by the Animal Review Committee at University of Michigan. Male C57BL/6J (9–10 weeks old) mice were purchased from the Jackson Laboratory (Bar Harbor, ME). Mice were housed for 3 days before any procedures.

### LPS-induced endotoxic shock model

Experiment Ι: Mice were injected intraperitoneally (i.p.) with YW3-56 (5 mg/kg, kindly provided by Dr. Yanming Wang, Department of Biochemistry and Molecular Biology, Penn State University, University Park, PA) dissolved in DMSO (Sigma Aldrich Inc., St. Louis, MO, USA) or vehicle DMSO 20 minutes after LPS (35 mg/kg). Mice receiving only DMSO served as controls (n = 8–12/group). Long term survival was monitored for 10 consecutive days. Experiment ΙΙ: Mice were randomly divided into 3 groups: (a) vehicle DMSO only group (DMSO); (b) LPS insult followed by vehicle DMSO treatment group (LPS); (c) LPS insult followed by YW3-56 treatment group (LPS + YW3-56). All reagents were given only once unless noted otherwise. Animals were sacrificed at 0.5, 3, 12 and 24 hours after treatments (6–8 mice per time point per group). Blood samples were collected at each time point and stored in a −80 °C freezer for further purposes.

### Hemorrhagic shock-induced model

Hemorrhagic shock was induced in a murine model as described previously^13^. Briefly, anesthesia was achieved with 5% isoflurane and maintained with 2% isoflurane. Bupivacaine (1%) was injected locally at the operative site. With aseptic technique, the femoral artery was cannulated with polyethylene 10 tubing (PE10, Clay Adams, Sparks, MD). Heart rate and blood pressure were monitored by Ponemah Physiology Platform (Ground Instrument Systems, Valley View, OH). Thirty percent blood loss was accomplished based on following formula: Estimated blood loss volume (ml) = body weight (g) × 0.07 (ml/g) × 30%. Animals from Sham group were not cannulated and not subjected to hemorrhage. Blood was collected 0.5, 3, 12 and 24 hours post hemorrhage. Serum was prepared and stored at −80 °C for further purposes.

### Development of “Sandwich” indirect ELISA

To quantify CitH3 in the blood, we developed a “sandwich” indirect ELISA. In brief, 0.2ug/well anti-CitH3 monoclonal antibody was coated onto 96-well plate as capture antibody overnight at 4 °C, then plates were blocked by protein-free blocking buffer (Thermo Scientific, Rockford, IL, USA) for 2 h at room temperature (RT). Serum was treated with DNase (150 unit/ml, Sigma Aldrich, St. Louis, MO, USA) for 1 hour at 37 °C with supplement of 1 mM calcium chloride. DNase treated serum (20 µl) was added to the wells with 80 µl blocking buffer and incubated at RT for 2 hours. After four washings, anti-CitH3 rabbit polyclonal antibody (1:3000 diluted, Abcam, Cambridge, MA, USA) was added as detecting antibody for 2 hours at RT. Following 4 times of washing, anti-rabbit peroxidase-labeled secondary antibody (1:50000 diluted, Jackson ImmunoResearch, West Grouve, PA, USA) was incubated in wells for 1 hour at RT. After 4 thorough washes to remove extra secondary antibodies, plate was developed with 3, 3′, 5, 5′-Tetramethylbenzidine (TMB) for 20 minutes in dark followed by stop solution (R&D Systems Inc., Minneapolis, MN, USA). Absorbance at 450 nm wavelength was determined. Synthesized CitH3 peptide (New England peptide, Gardner, MA, USA) was utilized to generate the standard curve.

### Cytokine and PCT measurement

Concentrations of IL-1 beta and IL-6 in serum were detected utilizing ELISA (R&D Systems Inc., Minneapolis, MN, USA). Concentrations of circulating PCT were measured by ELISA (LS Bio Inc., Seattle, WA, USA). All procedures were performed in accordance with the instructions.

### Western blotting

Serum was 1:1 diluted with normal saline before being denatured and loaded equally to sodium dodecyl sulfate polyacrylamide gel electrophoresis (SDS-PAGE) gels. Membranes were probed by anti-CitH3 polyclonal antibody (1:1000 diluted, ab5103, Abcam, Cambridge, MA, USA). Ponceau S staining was performed as loading control.

### Quantification of NETs

To quantify NET-DNA in mouse serums, a PicoGreen assay kit (Invitrogen, San Diego, CA, USA) was used according to manufacture’s instruction.

### Statistical Analysis

Results were presented as mean ± SD. Differences between multiple groups were assessed by one-way analysis of variance (ANOVA) followed by Bonferroni post hoc testing for multiple comparisons. Student’s t-test was performed to determine the differences between two groups. Kaplan-Meier method was used for survival analysis, and log-rank test was used to compare the difference in survival rates. Analysis was performed y GraphPad Prism (GraphPad Software Inc., La Jolla, CA, USA). *P* value of no more than 0.05 was considered significant.

## Electronic supplementary material

Supplementary Information

## References

[1] Mayr FB, Yende S, Angus DC (2014). Epidemiology of severe sepsis. Virulence.

[2] Singer M (2016). The Third International Consensus Definitions for Sepsis and Septic Shock (Sepsis-3). JAMA: the journal of the American Medical Association.

[3] Valparaiso AP, Vicente DA, Bograd BA, Elster EA, Davis TA (2015). Modeling acute traumatic injury. The Journal of surgical research.

[4] Neeli I, Khan SN, Radic M (2008). Histone deimination as a response to inflammatory stimuli in neutrophils. Journal of immunology.

[5] Konig MF, Andrade F (2016). A Critical Reappraisal of Neutrophil Extracellular Traps and NETosis Mimics Based on Differential Requirements for Protein Citrullination. Frontiers in immunology.

[6] Brinkmann V (2004). Neutrophil extracellular traps kill bacteria. Science.

[7] Clark SR (2007). Platelet TLR4 activates neutrophil extracellular traps to ensnare bacteria in septic blood. Nature medicine.

[8] Guimaraes-Costa AB, Nascimento MT, Wardini AB, Pinto-da-Silva LH, Saraiva EM (2012). ETosis: A Microbicidal Mechanism beyond Cell Death. Journal of parasitology research.

[9] Wang S, Wang Y (2013). Peptidylarginine deiminases in citrullination, gene regulation, health and pathogenesis. Biochimica et biophysica acta.

[10] Zhang X (2012). Peptidylarginine deiminase 2-catalyzed histone H3 arginine 26 citrullination facilitates estrogen receptor alpha target gene activation. Proceedings of the National Academy of Sciences of the United States of America.

[11] Wang Y (2009). Histone hypercitrullination mediates chromatin decondensation and neutrophil extracellular trap formation. The Journal of cell biology.

[12] Wang Y (2012). Anticancer peptidylarginine deiminase (PAD) inhibitors regulate the autophagy flux and the mammalian target of rapamycin complex 1 activity. The Journal of biological chemistry.

[13] Li Y (2011). Identification of citrullinated histone H3 as a potential serum protein biomarker in a lethal model of lipopolysaccharide-induced shock. Surgery.

[14] Li Y (2014). Citrullinated histone H3: A novel target for the treatment of sepsis. Surgery.

[15] Riedel S (2012). Procalcitonin and the role of biomarkers in the diagnosis and management of sepsis. Diagnostic microbiology and infectious disease.

[16] Pierrakos C, Vincent JL (2010). Sepsis biomarkers: a review. Critical care.

[17] Dandona P (1994). Procalcitonin increase after endotoxin injection in normal subjects. The Journal of clinical endocrinology and metabolism.

[18] Wacker C, Prkno A, Brunkhorst FM, Schlattmann P (2013). Procalcitonin as a diagnostic marker for sepsis: a systematic review and meta-analysis. The Lancet. Infectious diseases.

[19] Tang Y (2012). Establishment of an experimental mouse model of trauma-hemorrhagic shock. Exp Anim.

[20] Pfeifer R (2013). Models of hemorrhagic shock: differences in the physiological and inflammatory response. Cytokine.

[21] Papayannopoulos V, Zychlinsky A (2009). NETs: a new strategy for using old weapons. Trends in immunology.

[22] Yousefi S (2008). Catapult-like release of mitochondrial DNA by eosinophils contributes to antibacterial defense. Nature medicine.

[23] Fuchs TA (2007). Novel cell death program leads to neutrophil extracellular traps. The Journal of cell biology.

[24] Li P (2010). PAD4 is essential for antibacterial innate immunity mediated by neutrophil extracellular traps. The Journal of experimental medicine.

[25] Arita K (2006). Structural basis for histone N-terminal recognition by human peptidylarginine deiminase 4. Proceedings of the National Academy of Sciences of the United States of America.

[26] Millius A, Weiner OD (2009). Chemotaxis in neutrophil-like HL-60 cells. Methods in molecular biology.

[27] Stearns-Kurosawa DJ, Osuchowski MF, Valentine C, Kurosawa S, Remick DG (2011). The pathogenesis of sepsis. Annu Rev Pathol.

[28] Kibe S, Adams K, Barlow G (2011). Diagnostic and prognostic biomarkers of sepsis in critical care. The Journal of antimicrobial chemotherapy.

[29] Yipp BG, Kubes P (2013). NETosis: how vital is it?. Blood.

[30] Neeli I, Radic M (2013). Opposition between PKC isoforms regulates histone deimination and neutrophil extracellular chromatin release. Frontiers in immunology.

