# Supplementary material for: CitH3: a reliable blood biomarker for diagnosis and treatment of endotoxic shock
Source: Sci Rep. 2017 Aug 21;7:8972. doi: 10.1038/s41598-017-09337-4 (PMC5567134; doi:10.1038/s41598-017-09337-4)
Supplement: Supplementary file 1 — Supplementary Information [file 41598_2017_9337_MOESM1_ESM.pdf]

CitH3: a reliable blood biomarker for diagnosis and treatment of endotoxic shock

Baihong Pan[1,2], Hasan B. Alam[1], Wei Chong[3], Jim Mobley[4], Baoling Liu[1], Qiufang Deng[1,2], Yinjian Liang[1,3], Yanming Wang[5], Eric Chen[1], Tianbing Wang[6], Muneesh Tewari[7], and Yongqing Li[1*]

[1] Department of Surgery, University of Michigan Hospital, Ann Arbor, MI, USA

[2] Department of General Surgery, Xiangya Hospital, Changsha, Hunan, China

[3] The First Hospital, China Medical University, Shenyang, China

[4] Cayman Chemical, Ann Arbor, MI, 48108, USA

[5] Department of Biochemistry and Molecular Biology, Penn State University, University Park, PA, US.

[6] Department of Trauma and Orthopedic Surgery, Peking University People's Hospital, Beijing, China

[7]Department of Internal Medicine, University of Michigan, Ann Arbor, MI, USA

*Corresponding author:

Yongqing Li, MD, PhD

Email: yqli@med.umich.edu

Department of Surgery

University of Michigan Medical School

NCRC, Room 363N, Building 26

Plymouth Road, Ann Arbor, MI, USA

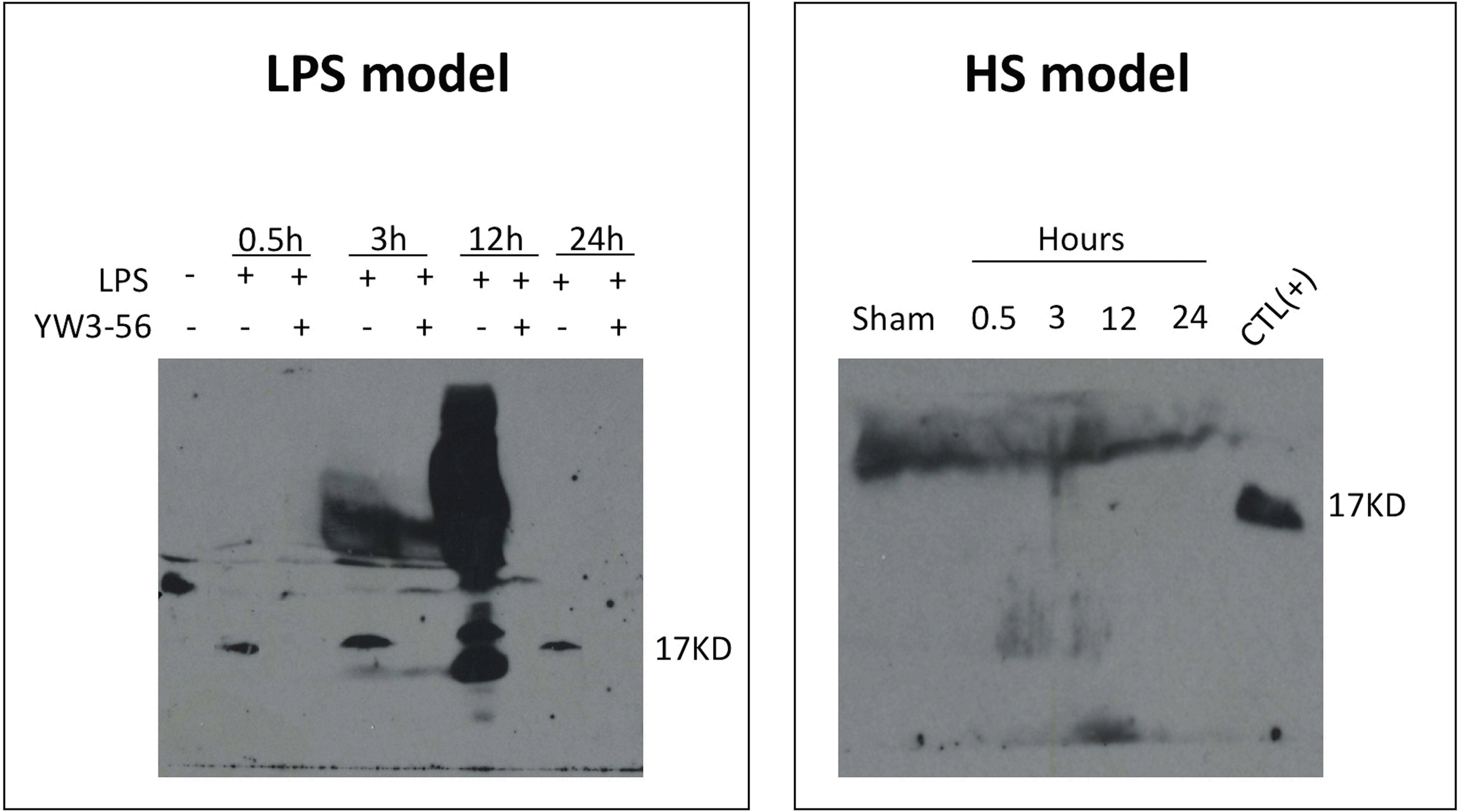
LPS model
0.5h
3h
12h
24h
LPS - + + + + + + + +
YW3-56 - - + - + - + - +
17KD
HS model
Hours
Sham
0.5
3
12
24
CTL(+)
17KD

Supplementary Figure 1. Effect of different treatments (LPS, LPS+YW3-56, or HS) on blood levels of CitH3 in mouse models of LPSS and HS. Full-length western blots of circulating CitH3 which corresponds to cropped blots presented in Figure 2 (A and C). See the figure legend of Figure 2 for detail.
